# The development of an immunoassay to measure immunoglobulin A in Asian elephant feces, saliva, urine and serum as a potential biomarker of well-being

**DOI:** 10.1093/conphys/coy077

**Published:** 2019-03-20

**Authors:** Katie L Edwards, Pakkanut Bansiddhi, Steve Paris, Marie Galloway, Janine L Brown

**Affiliations:** 1 Center for Species Survival, Smithsonian Conservation Biology Institute, 1500 Remount Rd., Front Royal, VA, USA; 2 Center of Excellence in Elephant and Wildlife Research, Faculty of Veterinary Medicine, Chiang Mai University, 155 Irrigation Canal Road, Mae Hia, Muang, Chiang Mai, Thailand; 3 Center for Animal Care Sciences, Smithsonian’s National Zoological Park, 3001 Connecticut Ave, NW, Washington, DC, USA

**Keywords:** Asian elephant, glucocorticoids, immunoassay, Immunoglobulin A, well-being

## Abstract

Additional measures of well-being would be beneficial to the management of a variety of species in human care, including elephants. Immunoglobulin A (IgA) is an immune protein associated with pathogen defense, which has been demonstrated to decrease during times of stress, and increase in response to positive stimuli. This paper describes the development and validation of an enzyme immunoassay (EIA) for the quantification of Asian elephant (*Elephas maximus*) IgA in feces, saliva, urine, and serum. Samples were collected weekly from four females for 6 months to assess IgA and glucocorticoid (GC) concentrations, establish relationships between these two biomarkers, and determine variability in IgA within and between individuals, and across sample types. IgA was quantified in all four sample types, although urinary concentrations were low and sometimes undetectable in individual samples. Concentrations were highly variable within and between individuals, with fecal, salivary and serum IgA, and fecal, salivary and urinary GCs all differing significantly across individuals. Contrary to previous findings, IgA and GC were generally not correlated. Serum IgA was less variable within individuals, with the exception of one female that experienced a brief illness during the study. However, marked inter-individual differences were still apparent. When data from all individuals were combined, fecal IgA was significantly predicted by salivary and urinary IgA; however, this relationship did not hold when individuals were analyzed separately. Analysis of a fifth female that exhibited a more severe systemic illness demonstrated clear increases in fecal IgA and GC, suggesting these may also be useful health biomarkers. Further investigation is needed to determine what sample type is most reflective of biological state in elephants, and how IgA concentrations are associated with health and positive and negative welfare states. Based on observed variability, a longitudinal approach likely will be necessary to use IgA as a measure of well-being.

## Teaser

Identifying biomarkers of well-being will be beneficial to our understanding of animal welfare. We developed and validated an assay for measuring Asian elephant immunoglobulin A (IgA) in feces, saliva, urine and serum as a potential welfare measure, and show that longitudinal assessments are necessary because of high intra-individual variability.

## Introduction

Modern zoos have a responsibility to maintain animals under the highest standards of care, the key to which is understanding species biology and natural history to ensure captive environments meet both physical and psychological needs. In recent years, a scientific approach to studying zoo elephant welfare has led to great strides in improving the care and management of African (*Loxodonta africana*) and Asian (*Elephas maximus)* elephants. Indeed, a recent epidemiological study in North America revealed a number of variables correlated with positive welfare outcomes (Carlstead *et al.*, 2013; Brown *et al.*, 2016; Miller *et al.*, 2016; Morfeld *et al.*, 2016; Prado-Oviedo *et al.*, 2016; Greco *et al.*, 2016a, 2016b; Holdgate *et al.*, 2016a, 2016b; Meehan *et al.*, 2016a, 2016b). Although several important factors were identified, these were primarily population-level results, making it difficult to assess individual well-being. Despite improved understanding of elephant physiology over the last three decades, significant health (Fowler and Mikota, 2006) and reproductive (Brown, 2014) issues remain, so additional measures to assess physiological state would be beneficial to species management.

Traditional welfare assessment methods have focused primarily on negative states, such as the occurrence of abnormal behaviors, poor health and survival, the lack of reproductive function, or elevated stress hormones (glucocorticoids, GC) (Broom, 1991). Glucocorticoid measures can be a useful marker of physiological state, especially when assessed non-invasively (Schwarzenberger, 2007), but need to be interpreted correctly. Increases in concentrations are associated with acute (Scheiber *et al.*, 2005; Viljoen *et al.*, 2008; Voellmy *et al.*, 2014) and chronic (Gobush *et al.*, 2008; Blickley *et al.*, 2012; Parry-Jones *et al.*, 2016) stress, but also can occur in animals coping appropriately with day-to-day challenges, including positive stimuli such as pleasure, excitement and arousal (Ralph and Tilbrook, 2016). They may also reflect normal physiological states; e.g. during the estrous cycle (Fanson *et al.*, 2014) and pregnancy (Kersey *et al.*, 2011; Marciniak *et al.*, 2011). Indeed, individuals may be more or less responsive to potential challenges due to different coping styles (Curley *et al.*, 2008; Koolhaas, 2008). This normal variation must be taken into account when using GCs as a welfare measure, necessitating longitudinal analyses to reliably understand biological relevance. Although these measures are still of great importance, attention has turned more recently to finding additional markers of well-being, including those that indicate positive affect (Yeates and Main, 2008). Incorporating measures of both positive and negative states allows an evaluation of welfare as a continuum, assessing factors that are good for an individual, as opposed to just not being bad.

Biomarkers of immune function have previously been used to assess welfare, because stress can have immunosuppressive effects (Siegel, 1987). For example, cell-mediated and humoral immune responses were influenced by housing condition and stocking density of ewes (Caroprese *et al.*, 2008), and alterations in biomarkers of the innate immune response and acute phase reaction were associated with potentially stressful changes in housing of pigs (Marco-Ramell *et al.*, 2016). Another potential biomarker of well-being is immunoglobulin A (IgA) (Staley *et al.*, 2018), an antibody that plays an important role in the immune defense against pathogens. There are typically two forms of IgA, which differ both in structure and in function (Kerr, 1990). Secretory IgA exists as a dimer that also contains a J-chain and a secretory component to protect against proteases. This form is produced at mucosal linings, and is present in saliva, tears, bile, milk and mucosal secretions of the reproductive, respiratory and gastrointestinal systems (Pihl and Hau, 2003), where it acts as the first defense against pathogens including viruses and bacteria. Monomeric IgA is found in serum, produced by plasma cells in the bone marrow and acts as a secondary line of defense to eliminate pathogens that breach the mucosal surface (Woof and Kerr, 2004). Due to the abundance of IgA secreting cells in normal mucosa, IgA comprises at least 70% of immunoglobulins produced in mammals (Macpherson *et al.*, 2008). In addition to being an indicator of immune function, IgA has been shown to decrease during times of stress. Physical stressors such as intensive exercise (Gleeson *et al.*, 1995; Skandakumar *et al.*, 1995), psychological challenges (Deinzer and Schuller, 1998; Ng *et al.*, 1999), metabolic demand (Royo *et al.*, 2005), and relocation to a new environment (Bundgaard *et al.*, 2012) have all been associated with decreased IgA. Interestingly, however, IgA has also been shown to increase in response to positive stimuli, such as relaxation and positive emotional states (Green *et al.*, 1988), and so has been suggested to be a potential marker of positive well-being (Yeates and Main, 2008).

A further advantage to the use of IgA is that it can be measured in multiple biological samples, including serum (Maes *et al.*, 1997; Mishra *et al.*, 2011; Moazzam *et al.*, 2013), saliva (Kikkawa *et al.*, 2003; Lucas *et al.*, 2007; Kvietkauskaite *et al.*, 2014), urine (Eriksson *et al.*, 2004; Rehbinder and Hau, 2006; Paramastri *et al.*, 2007), and feces (Royo *et al.*, 2004; Rehbinder and Hau, 2006; Paramastri *et al.*, 2007). Although IgA has been measured in a variety of species, including cats, dogs, humans, pigs, primates, reindeer and rodents, studies often are limited to either a single sample type or a limited number of samples over time. Immunoglobulin A production and secretion is tightly controlled at the local level, and influenced by physiological signals including those associated with immune and stress responses (see Staley *et al.* (2018) for a review), meaning concentrations may be variable both between sample types and over time. Past research has highlighted inconsistencies in the IgA response to acute stressors (Staley *et al.*, 2018), perhaps because of differences in the prior state of the individual or in the type of response required to deal with the stressor involved. Furthermore, acute stressors may be associated with increases in IgA, whereas chronic stress may be associated with decreased IgA (Staley *et al.*, 2018). For IgA to be a useful physiological biomarker of animal well-being, it is imperative to determine the degree of within and between individual variability, and to understand how acute or chronic challenges may impact IgA concentrations. The goal of this study was to develop an enzyme immunoassay (EIA) to measure IgA in multiple biological sample types, specifically feces, saliva, urine and serum in Asian elephants. We then set out to compare concentrations of IgA and GCs concurrently to investigate relationships between these two biomarkers across multiple sample types and over time, as a first step to determining if IgA can be a useful marker to assess well-being in this species.

## Methods

### Animals and sample collection

Samples were collected over a 6-month period from four female Asian elephants at the Smithsonian’s National Zoological Park, designated A–D, which were 69, 42, 42 and 27 years of age, respectively. This research was approved by the Animal Care and Use Committee of the Smithsonian National Zoological Park and Conservation Biology Institute (NZP-ACUC #15-03). Blood was collected from an ear vein as part of the weekly management routine, allowed to clot at room temperature (RT), centrifuged, and the serum harvested. Saliva was collected on the same day as serum, using a Cortisol-Salivette^®^ system (Sarstedt Inc., Newton, NC). Urine was collected opportunistically, generally free-catch, and typically on the same day as serum and saliva, or within 1–2 days. Feces were collected the day following serum and saliva collection, to allow for an estimated gut transit excretion rate of 24 h in this species (Fuller *et al.*, 2011; Edwards *et al.*, 2015). In addition, fecal samples collected surrounding a significant health event was analyzed in a fifth elephant (E; 39 years of age). All samples were frozen –20°C until analysis.

### Fecal extraction

For analysis of fecal IgA, feces were dried in a lyophilizer, sifted to remove fibrous material, weighed 0.1000 g (±0.0010g) and added to 3 ml of phosphate buffered saline with Tween (PBS-T; 0.01 M phosphate buffer, 0.50 M NaCl, 0.1% Tween 20^®^, pH 7.2). Samples were vortexed thoroughly to ensure free-mixing of the fecal powder, and agitated overnight on a multi-tube pulse vortexer (Glas-Col, Terre Haute, IN). Samples were then vortexed briefly and centrifuged at 1800 ×* g* for 20 min at 4°C to pellet fibrous material. The supernatant was decanted into a clean tube and centrifuged again at 3500 × *g* for 10 min at 4°C to pellet the particulate. From this, 2.0 ml of supernatant was removed, evaporated to dryness under air, re-suspended in 0.5 ml ultra purified water, and stored at −20°C until analysis.

For analysis of fecal GC, fecal samples were processed using a dry-weight shaking extraction technique adapted from Scarlata *et al.* (2011). In brief, 0.1000 g (±0.0010 g) lyophilized fecal powder was added to 5.0 ml of 80% methanol. Samples were vortexed and agitated on a multi-tube pulse vortexer for 30 min, before being centrifuged at 1500 × *g* for 20 min. Supernatants were decanted before a further 5.0 ml of 80% methanol was added to the original tubes containing the fecal pellets, vortexed, and centrifuged again (1500 × *g* for 15 min). Combined supernatants were evaporated to dryness before being re-suspended in 1.0 ml 100% methanol. Extracts were dried again before final re-suspension in 1 ml phosphate buffer (0.039 M NaH_2_PO_4_, 0.061 M Na_2_HPO_4_, 0.15 M NaCl; pH 7.0), and stored frozen at −20°C until analysis. The average extraction efficiency of this process was 86.3% (range 77.9–99.8%) based on addition of ^3^H-corticosterone to each sample prior to extraction.

### Immunoassays

Immunoglobulin A was quantified in Asian elephant feces, saliva, urine and serum by EIA using commercially available components. A polyclonal rabbit anti-human IgA antibody (A0262, Dako, Glostrup, Denmark) was diluted to a working concentration of 10 mg/l in phosphate buffered saline (0.01 M phosphate buffer, 0.15 M NaCl, pH 7.2) and 100 μl added per well to a 96-well microtiter plate (Costar, Corning Life Sciences, Tewkesbury, MA). After incubation overnight at 4°C, plates were aspirated and washed three times with PBS-T. Standards (0.39–100 ug/l), high and low concentration controls made using IgA from human colostrum (I2636, Sigma Aldrich, St. Louis, MO), and biological samples diluted as necessary in PBS-T (fecal extract: 1:20 to 1:500; saliva: 1:250; urine: neat to 1:20; serum: 1:500 to 1:5000) were added in duplicate (50ul). Following incubation at room temperature (RT) for 2 h on a plate shaker set to 500 RPM, plates were aspirated and washed three times with PBS-T. A polyclonal rabbit anti-human IgA antibody conjugated to horseradish peroxidase (HRP; P0216, Dako, Glostrup, Denmark) was diluted 1:2000 in PBS-T and 100 ul added per well before incubation at RT for 1 h on a plate shaker set to 500 RPM. After a final (3x) wash step, 100 μl high kinetic 3,3’,5,5’-tetramethylbenzidine (TMB) peroxidase substrate (Moss Inc., Pasadena, MD) was added per well and incubated in the dark for 10 min at RT. Finally, the reaction was stopped with 50 μl stop solution (1N HCl) and the absorbance measured at 450 nm with a reference of 570 nm using a microplate reader (Filtermax F5, Molecular Devices, Sunnyvale, CA).

The IgA antibodies cross-react with the alpha-chains of human IgA, and do not cross-react with human IgG or IgM. According to Humphreys *et al.* (2015), the predicted structure of Asian elephant IgA is very similar to that of human, supporting the use of these antibodies that have previously been demonstrated to cross-react with IgA in other species including cow, deer, goat, horse, mink, mouse, polecat, sheep and swine (Hau *et al.*, 1990). The IgA EIA was validated biochemically for Asian elephant fecal extract, saliva, urine and serum through parallelism and matrix interference assessment, and subsequent regression analyses. Serial sample dilutions yielded displacement curves parallel to the standard curve (feces: *y *= 0.999*x* + 0.183, *R*^2^ = 0.952, *F*_1,6_ = 120.105, *P* < 0.001; saliva: *y* = 0.983*x* + 0.046, *R*^2^ = 0.968, *F*_1,6_ = 181.866, *P* < 0.001; urine: *y *= 1.503*x* − 0.009, *R*^2^ = 0.999, *F*_1,5_ = 9066.849, *P* < 0.001; serum: *y* = 0.764*x*+ 0.071, *R*^2^ = 0.926, *F*_1,6_ = 75.430, *P* < 0.001). There was no evidence of matrix interference, as addition of each sample type to assay standards did not alter the amount observed (feces: *y* = 0.944*x* + 0.089, *R*^2^ = 1.000, *F*_1,6_ = 16622.520, *P* < 0.001; saliva: *y *= 0.907*x* − 0.434, *R*^2^ = 0.999, *F*_1,6_ = 6941.360, *P* < 0.001; urine: *y *= 1.062*x* − 0.029, *R*^2^ = 0.999, *F*_1,7_ = 10916.993, *P* < 0.001; serum:* y* = 0.982*x* − 0.704, *R*^2^ = 0.999, *F*_1,7_ = 9110.310, *P* < 0.001).

Glucocorticoids were measured using three different assays for the four sample types, according to assay validation results. Fecal GC metabolites were measured using a double antibody EIA incorporating a secondary goat-anti rabbit IgG antibody (A009, Arbor Assays, Ann Arbor, MI) and polyclonal rabbit anti-corticosterone antibody (CJM006, C. Munro, University of California, Davis, CA) adapted from Munro and Stabenfeldt (1984) and validated for Asian elephants by Watson *et al.* (2013). In brief, secondary antibody (150 μl; 10 μg/ml in coating buffer [X108, Arbor Assays]) was added to 96-well microtiter plates (Costar, Corning Life Sciences, Tewkesbury, MA) followed by incubation at RT for 15–24 h. After incubation, unbound antibody was washed from wells with wash buffer (X007, Arbor Assays). Blocking solution (250 μl; X109, Arbor Assays) was added to each well and left to incubate for 4–24 h at RT. Blocking solution was then removed and plates were dried at RT in a desiccator cabinet, packaged in vacuum-sealed bags, and stored at 4°C until use. Corticosterone standards (50 μl; 0.078–20 ng/ml), controls (50 μl), and samples (50 μl; diluted 1:10 in phosphate buffer [0.039M NaH_2_PO_4_, 0.061M Na_2_HPO_4_, 0.15M NaCl; pH 7.0]) were added to plate wells in duplicate. Corticosterone-HRP (25 μl; 1:25 000; C. Munro, University of California, Davis, CA) was added to all wells. The primary anti-corticosterone antibody (25 μl; CJM006 1:60 000) was added to all wells except for the non-specific binding (NSB) wells, followed by incubation for 2 h at RT. Unbound components were removed by washing five times with wash buffer (X007, Arbor Assays), followed immediately by the addition of a chromagen solution containing TMB (100 μl, X019, Arbor Assays) to each well. After incubation for 30 min at RT, the reaction was halted by the addition of stop solution (50 μl; X020 Arbor Assays) and optical densities were determined at 450 nm with a reference of 630 nm.

Salivary and urinary GC concentrations were quantified by a double antibody EIA using a secondary goat-anti rabbit IgG antibody (A009, Arbor Assays) and polyclonal rabbit anti-cortisol antibody (R4866, C. Munro, University of California, Davis, CA). The assay protocol was the same as described above for the corticosterone EIA, with the following alterations: cortisol standards (50 μl; 0.078–20 ng/ml), controls (50 μl), and samples (50 μl; diluted 1:2 to 1:4 [saliva] or 1:20 to 1:100 [urine] in phosphate buffer) were added in duplicate; cortisol-HRP (25 μl; 1:15 000; C. Munro, University of California, Davis, CA) was used in addition to the primary anti-cortisol antibody (25 μl; R4866 1:60 000); and assays were incubated at RT for 1 h before addition of TMB, and for 5 min before addition of stop solution. Urinary cortisol and IgA concentrations were indexed by creatinine (CRT) concentration according to Monfort *et al.* (1990).

Serum cortisol was measured using a solid-phase ^125^I radioimmunoassay (RIA) (Corti-Cote, MP Biomedicals, Santa Ana, CA) with some modifications. In brief, 25 μl of each calibrator, control, and sample were added in duplicate to pre-coated tubes containing cortisol antiserum. 250 μl of ^125^I-labeled cortisol tracer solution was added, tubes were mixed briefly and incubated for 45 min in a water bath at 37°C. Tubes were decanted thoroughly before being counted in a gamma counter (Iso Data 20/20 series).

The cross-reactivities of the antibodies CJM006 and R4866 have been reported elsewhere (Young *et al.*, 2004; Watson *et al.*, 2013). Cross reactivities for the cortisol RIA are as follows: cortisol 100.0%, prednisolone 94.1%, prednisone 1.2%, cortisone 0.8%, 17-hydroxyprogesterone <0.05%, corticosterone 1.2%, metyrapone <0.01%, dexamethasone 0.8%, and 11-deoxycortisol 2.2%. The immunoassays were validated biochemically for measuring GCs in fecal extracts, saliva, urine and serum through parallelism and matrix interference assessment, and subsequent regression analyses. Serial dilutions of Asian elephant samples yielded displacement curves parallel to the standard curve (feces: *y* = 1.306*x* − 3.372, *R*^2^ = 0.990, *F*_1,7_ = 704.673, *P* < 0.001; urine: *y* = 0.979*x* + 6.581, *R*^2^ = 0.992, *F*_1,6_ = 733.442, *P* < 0.001; saliva: *y* = 1.013*x* − 2.423, *R*^2^ = 0.996, *F*_1,5_ = 1339.416, *P* < 0.001; serum: *y* = 0.877*x* + 1.682, *R*^2^ = 0.993, *F*_1,4_ = 558.645, *P* < 0.001). There was no evidence of matrix interference, as addition of each sample type to assay standards did not alter the amount observed (feces: *y* = 1.231*x*− 0.389, *R*^2^ = 0.999, *F*_1,4_ = 4502.016, *P* < 0.001; urine: *y* = 1.033*x* − 6.473, *R*^2^ = 0.998, *F*_1,7_ = 3512.321, *P* < 0.001; saliva: *y* = 0.859*x* + 1.086, *R*^2^ = 0.999, *F*_1,7_ = 4977.480, *P* < 0.001; serum: *y* = 1.129*x* + 0.102, *R*^2^ = 0.998, *F*_1,6_ = 3696.085, *P* < 0.001). Inter and intra-assay coefficients of variation (CVs) were maintained below 15% and 10%, respectively, for all assays and sample types.

### Statistical analyses

Concentrations of IgA and GCs measured in each sample type were compared across the four elephants using generalized linear mixed models (GLMM) with individual as a random effect to account for non-independence of data. Potential relationships between IgA and GC concentrations within a sample type were compared using GLMMs, either by individual (sample date as random effect) or with all females combined (individual and sample date as random effects). Similarly, relationships between IgA concentrations measured in the four sample types were compared by individual (sample date as random effect) and with all females combined (individual and sample date as random effects). Data were log_10_ transformed where necessary to improve distribution, and GLMMs were performed in MLwiN version 2.02 (Rasbash *et al.*, 2005) using a normal distribution. Significance of the fixed effects was determined using a Wald test with chi-squared distribution (χ^2^), with alpha set to 0.05.

## Results

**Figure 1:**
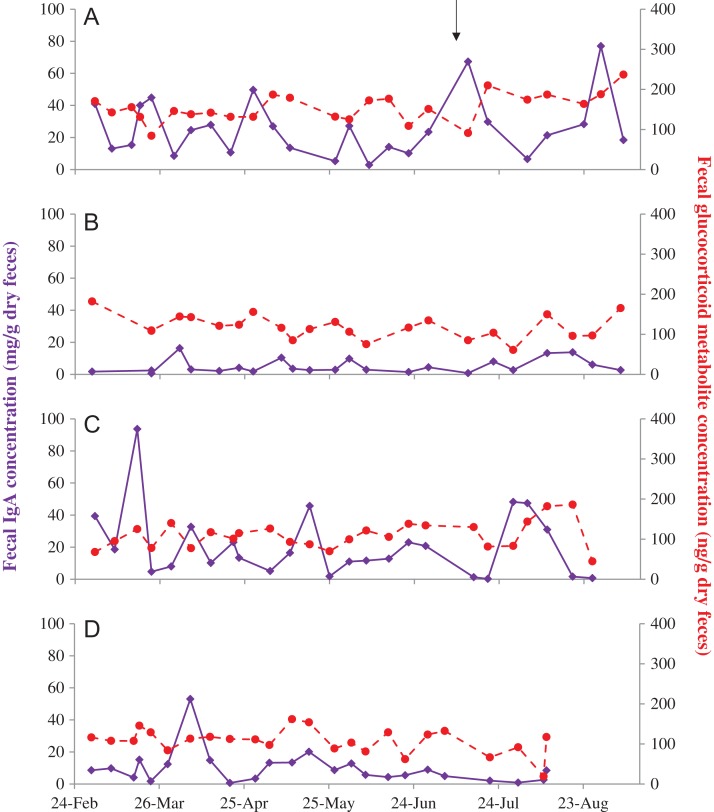
Fecal IgA (solid purple) and glucocorticoid metabolite (dashed red) concentrations in four female Asian elephants (**A–D**) aged 69, 42, 42 and 27 years, respectively. A brief illness in female A is denoted by the black arrow.

**Figure 2:**
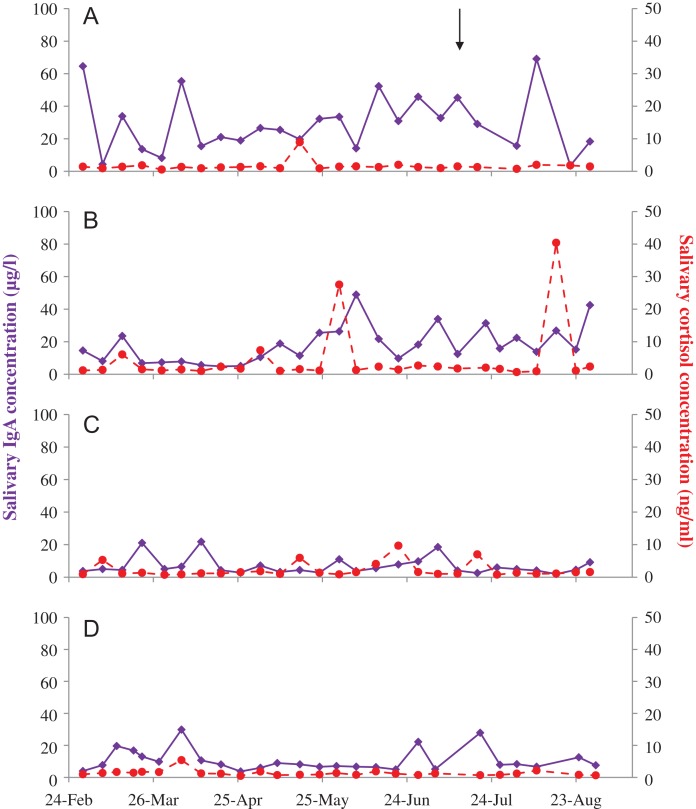
Salivary IgA (solid purple) and cortisol (dashed red) concentrations in four female Asian elephants (**A–D**) aged 69, 42, 42 and 27 years, respectively. A brief illness in female A is denoted by the black arrow.

**Figure 3:**
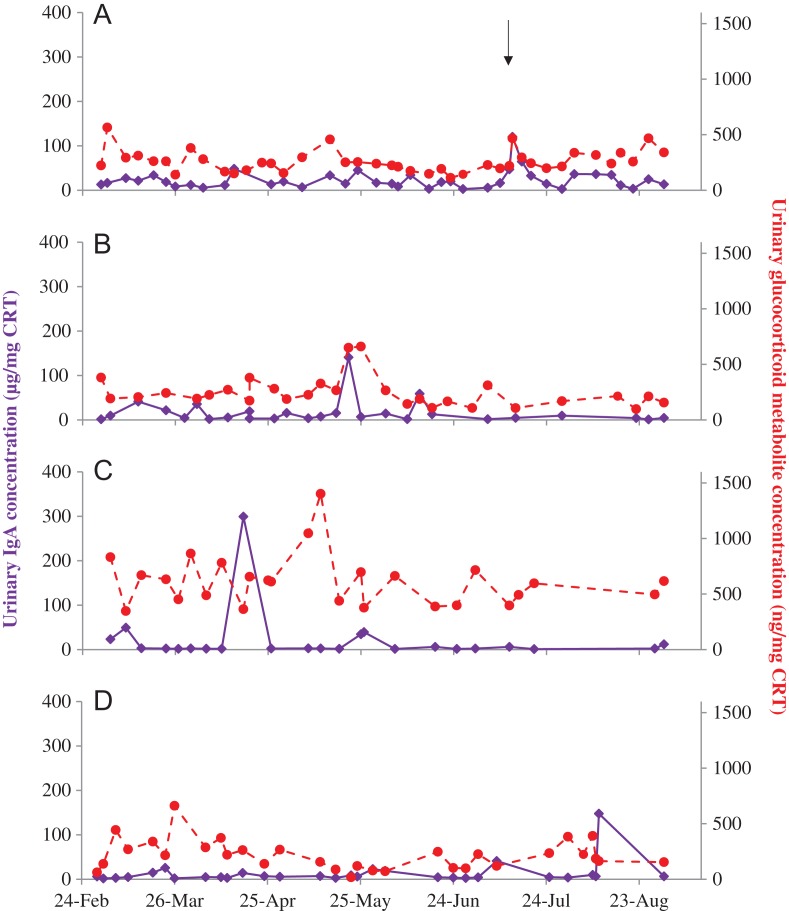
Urinary IgA (solid purple) and glucocorticoid metabolite (dashed red) concentrations in four female Asian elephants (**A–D**) aged 69, 42, 42 and 27 years, respectively. A brief illness in female A is denoted by the black arrow.

**Figure 4:**
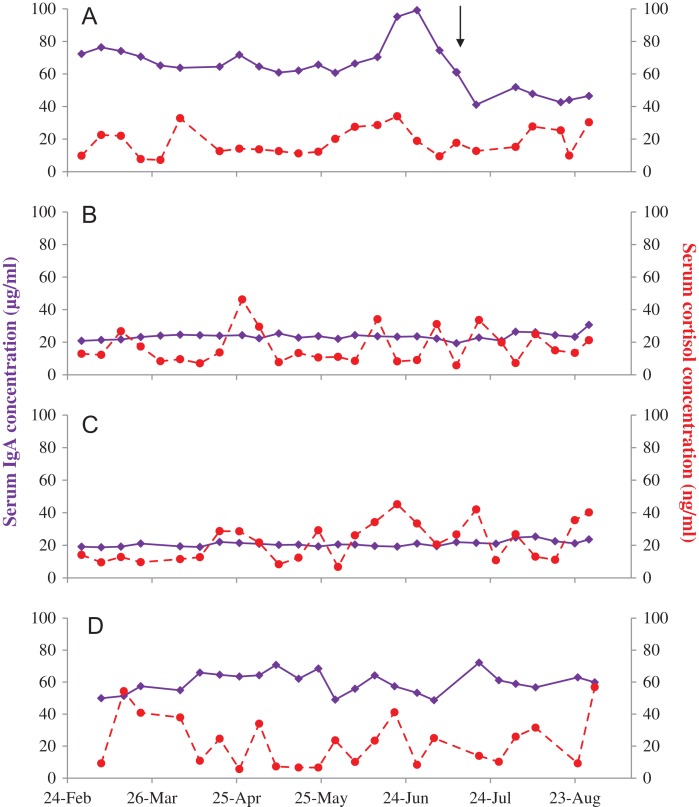
Serum IgA (solid purple) and cortisol (dashed red) concentrations in four female Asian elephants (**A–D**) aged 69, 42, 42 and 27 years, respectively. A brief illness in female A is denoted by the black arrow.

Immunoglobulin A was successfully quantified in all four sample types, although urinary IgA was only detected in 32–95% of samples within an individual; the remaining samples were below the detection limit of the assay. IgA and GC concentrations in feces, saliva, urine and serum are summarized in Table 1. There was considerable intra-individual variability in both IgA and GC concentrations across the four sample types (Figs 1–4). With the exception of urinary IgA and serum cortisol, concentrations also varied significantly among the four females (Table 1). Fecal IgA was higher in females A and C compared to D and B (Table 1, Fig. 1). Fecal GC metabolite concentrations were also higher in female A compared to the other three females. However, fecal IgA and GC metabolite concentrations were not related in any of the four females individually (*P* > 0.122), or when all data were combined (*P* = 0.229).

**Table 1:** Mean, range, standard deviation (SD), coefficient of variation (CV) and number of samples quantified for glucocorticoid (GC) and immunoglobulin A (IgA) concentrations in four female Asian elephants (A–D) aged 69, 42, 42 and 27 years, respectively

	Elephant				GLMM		
	A	B	C	D	χ^2^	df	*P*
Fecal IgA (mg/g dry feces)							
*N*	26	23	25	24	31.946	3	**<0.001**
Mean	25.9^b^	5.1^a^	20.9^b^	9.8^a^			
Range	2.9–77.0	0.6–16.3	0.2–93.7	0.7–53.0			
SD	18.4	4.5	21.4	10.6			
CV	71.1	88.7	102.7	107.9			
Fecal GC (ng/g dry feces)							
Mean	154.2^b^	118.4^a^	109.6^a^	107.1^a^	21.779	3	**<0.001**
Range	84.2–236.8	61.0–182.0	44.5–186.2	19.0–161.8			
SD	34.6	29.6	34.2	31.1			
CV	22.4	25.0	31.2	29.0			
Salivary IgA (μg/l)							
*N*	25	27	27	55	77.710	3	**<0.001**
Mean	29.2^d^	18.1^c^	6.9^a^	11.3^b^			
Range	3.5–69.1	4.9–48.9	1.9–21.8	3.7–30.0			
SD	17.8	11.5	5.4	6.8			
CV	61.1	63.5	78.3	60.6			
Salivary GC (ng/ml)							
Mean	1.6^a^	4.3^b^	2.2^ab^	1.4^a^	11.932	3	**0.008**
Range	0.5–8.9	0.7–40.4	0.7–9.7	0.4–5.4			
SD	1.6	8.8	2.2	0.8			
CV	97.4	204.1	103.4	55.9			
Urinary IgA (μg/mg CRT)							
*N*^†^	40	20	9	10	1.861	3	0.602
Mean	23.3	21.6	52.3	30.0			
Range	2.7–120.3	1.8–140.8	1.0–299.2	3.7–147.8			
SD	21.4	31.6	94.1	42.9			
CV	91.8	146.2	180.0	142.9			
Urinary GC (ng/mg CRT)							
*N*^†^	42	29	26	31	97.618	3	**<0.001**
Mean	259.9^b^	244.9^ab^	617.0^c^	217.1^a^			
Range	111.8–565.2	97.0–660.3	347.3–1402.2	17.9–661.0			
SD	98.0	136.0	237.4	135.3			
CV	37.7	55.5	38.5	62.3			
Serum IgA (μg/ml)							
*N*	25	27	26	57	1643.846	3	**<0.001**
Mean	64.5^c^	23.5^b^	20.9^a^	62.5^c^			
Range	41.1–99.1	19.3–30.6	18.8–25.3	48.7–116.1			
SD	14.3	2.1	1.7	10.6			
CV	22.2	9.1	8.2	17.0			
Serum GC (ng/ml)							
Mean	18.2	17.0	22.0	23.4	4.529	3	0.210
Range	7.2–34.0	5.8–46.2	6.7–45.2	5.4–72.0			
SD	8.3	10.5	11.7	15.6			
CV	45.5	62.0	53.3	66.7			

^a,b,c,d^Significant difference between females within a sample type.

^†^The difference in *N* between urinary IgA and urinary GC reflects the number of samples per individual that were undetectable for IgA.

Salivary IgA exhibited high inter-individual variability, being highest in female A, followed by B, then D, and was lowest in female C (Table 1, Fig. 2; all comparisons *P* ≤ 0.009). Salivary GC was highest in female B, significantly higher than females A and D (Table 1). IgA and GC concentrations in saliva were not related in any of the four females individually (*P* > 0.121), or combined (*P* = 0.111).

Urinary IgA did not differ statistically among the four females, with concentrations reasonably stable over the study period (Table 1, Fig. 3). GC concentrations were also relatively low and stable in the urine of three of the females, but significantly higher in female C. There was no correlation between urinary IgA and GC when samples from all four females were combined (*P* = 0.460), or for three of the females individually (B–D, *P* > 0.155). However, in female A there was a positive relationship between IgA and GC (χ^2^ = 4.378, df = 1, *P* = 0.036).

Serum IgA was generally less variable within individuals over the study period, with the exception of female A (Fig. 4). This individual exhibited mild clinical signs of illness including lethargy and anorexia for a few days in mid-July. This illness was preceded by an increase in serum IgA concentrations, which subsequently decreased to below her typical concentrations at the time clinical signs were apparent. Female D, who exhibited no clinical signs during the study period, and Female A both had serum IgA concentrations around 3-fold higher than the other two females (Table 1). Serum cortisol did not differ among the four females, and IgA and cortisol were not significantly related, either when all data were combined (*P* = 0.350), or within each individual (*P* > 0.318).

**Figure 5:**
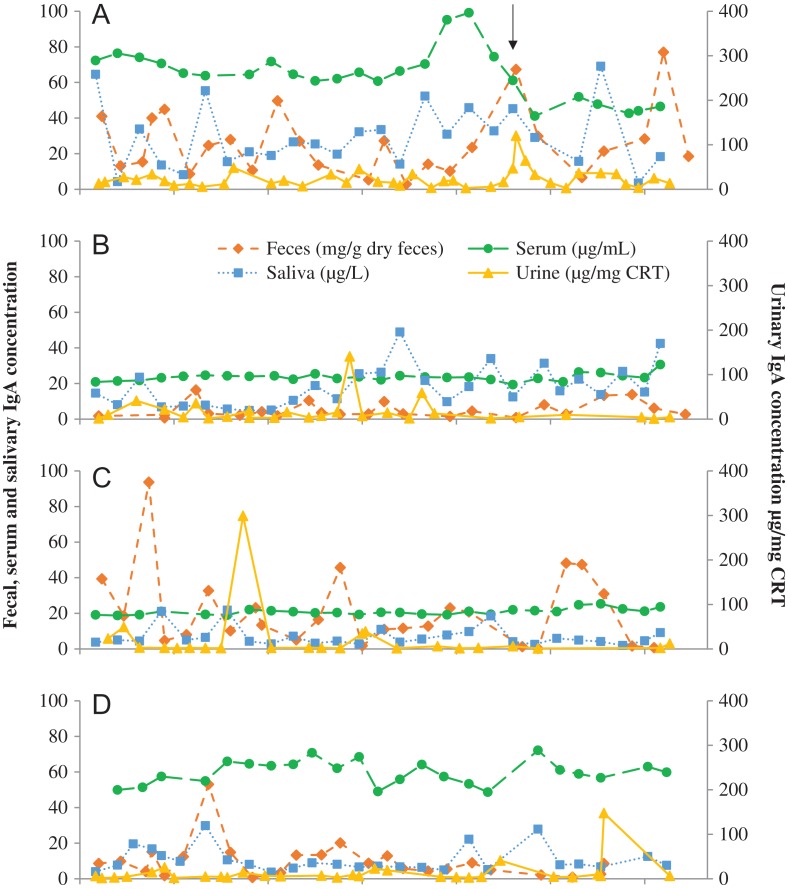
IgA measured across four sample types, feces (dashed orange), serum (dashed green), saliva (dotted blue) and urine (solid yellow) in four female Asian elephants (**A–D**) aged 69, 42, 42 and 27 years, respectively. A brief illness in female A is denoted by the black arrow.

Temporal patterns in IgA across the four sample types were generally not well correlated within-individual females (Fig. 5). Although there were some significant relationships within individuals, these tended to be when concentrations were not very variable across the study period. In female B, serum IgA was a significant predictor of fecal IgA (χ^2^ = 6.130, df = 1, *P* = 0.013), and in female C, urinary IgA was significantly related to salivary IgA (χ^2^ = 7.780, df = 1, *P* = 0.005) and serum IgA (χ^2^ = 10.111, df = 1, *P* = 0.001). It should be noted, however, that the number of urine samples with detectable concentrations of IgA was limited in this individual. In all other cases, IgA concentrations within one sample type were not correlated with other sample types. When samples from all females were combined, fecal IgA concentrations were positively correlated with both salivary IgA (χ^2^ = 4.137, df = 1, *P* = 0.042) and urinary IgA (χ^2^ = 8.174, df = 1, *P* = 0.004) concentrations.

**Figure 6:**
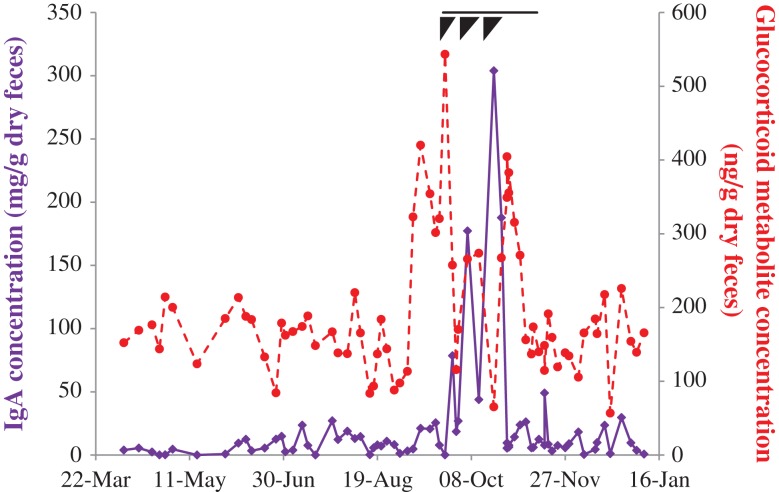
Fecal IgA (solid purple) and glucocorticoid metabolite (dashed red) concentrations in a 39 year old Asian elephant that experienced a severe bout of illness including lethargy, inappetance, abdominal distension and other idiopathic signs of discomfort. The total duration of clinical signs associated with this illness is denoted by the black line; black triangles denote three periods of peak clinical signs that subsided prior to the next recurrence.

Fecal IgA and GCs from Female E during a severe health event are presented in Fig. 6. Although no definitive diagnosis was made, the female presented with lethargy, inappetence, abdominal distension and other idiopathic signs of discomfort. The episode, thought to be a systemic infection, lasted around 7 weeks in total, with three more severe bouts during the initial 3 weeks. Fecal GCs showed a 4-fold increase over baseline concentrations, beginning 15 days prior to the onset of clinical signs, and lasting until all clinical signs had resolved. Interestingly, fecal IgA also increased ~30-fold, peaking around the end of the third more severe bout, and in association with more acute clinical signs. Although both fecal IgA and GCs increased during this period, the two were not significantly correlated (χ^2^ = 1.048, df = 1, *P* = 0.306).

## Discussion

Additional physiological measures of well-being, including those that represent both positive and negative affect, are required to help us better assess animal welfare. Immunoglobulin A has been proposed as such a measure (Yeates and Main, 2008; Staley *et al.*, 2018), and here we validate the use of an EIA to detect IgA in Asian elephant feces, saliva, urine and serum, and compare concentrations to respective GC measures. Our results demonstrate high variability in this protein both between and within individuals over time, and between different sample types, all of which have implications for future investigations. However, unlike some previous studies, where IgA has been shown to negatively correlate with GCs, the two were not significantly related in this study. With the exception of two examples of illness (one mild and one severe), we did not examine specific stressors during this study. Rather, the goal was to examine how IgA varied both within and between individuals, information that is important for understanding confounds related to using this biomarker as a potential measure of well-being.

Other species have shown high inter- and intra-individual variation in IgA excretion (Paramastri *et al.*, 2007). Royo and colleagues (2004) found there was about a 10-fold difference in fecal IgA between rats with the highest and lowest concentrations. Similarly, mean IgA concentrations varied among individual chimpanzees, tending to be higher in mature compared to immature individuals (Lantz *et al.*, 2016), and both age and sex-related differences were reported in reindeer (Yin *et al.*, 2015). The four females in this study differed widely in age, and the oldest elephant had the highest mean IgA concentrations in feces, saliva and serum; however, patterns among the other three individuals were not as consistent and overall age-related differences were not apparent. This suggests that age alone cannot explain all of the inter-individual variability observed here. In a previous study by Bundgaard and colleagues (2012), fecal IgA concentrations in mice followed a bimodal distribution, with distinct groups of high and low excretion. In the 6 weeks following the transfer of mice to a novel environment, the majority of high-excreting animals had switched to being low-excreters, without any intermediate states. All animals were from the same age cohort, and the same distribution was true for both males and females, so there must be some other explanation for the two non-overlapping groups. In addition to inter-individual variability in IgA concentrations, within-individual variation can be influenced by external factors. Both diurnal and/or seasonal differences in IgA measures have been observed in chimpanzees (Lantz *et al.*, 2016) and Sichuan golden monkeys (Huang *et al.*, 2014), and so should be taken into consideration when exploring changes in IgA in relation to health and welfare status.

Analysis of IgA in fecal extracts, saliva, urine and serum over the same period revealed that similar trends were not always apparent. Serum IgA has a different structure and function to that of secretory IgA (Kerr, 1990), as would be found in feces, saliva and urine, so a lack of similarity in profiles is perhaps not surprising. Serum IgA is produced by plasma cells in the bone marrow and acts as a secondary line of defense to eliminate pathogens that breach the mucosal surface (Woof and Kerr, 2004). However, in contrast to secretory IgA, the role of serum IgA in health and welfare remains relatively unexplored (Leong and Ding, 2014). With the exception of the brief illness in female A, serum IgA was generally less variable within individuals over the course of this study, but did reveal inter-individual variation that warrants further investigation. By contrast, secretory IgA in feces, saliva and urine is produced locally by plasma cells at mucosal linings to prevent invasion of inhaled and ingested pathogens, and it is this form of IgA that has been previously proposed as a potential welfare measure. When secretory IgA data were compared using fecal, saliva and urine samples within the same week, both saliva and urine were predictive of fecal concentrations, suggesting there is some similarity in excretion rates among the three routes. However, this did not hold true within individuals, so perhaps this overall relationship is reflective of similarities in the relative concentrations across the three sample types for each female, rather than between repeated samples within an individual over time. These data suggest that urine may not be the best measure of IgA in elephants due to the relatively low concentrations observed, with only around a third of samples quantifiable in one individual. Feces and saliva on the other hand generally had both higher and more variable concentrations of IgA throughout the study. Further investigation is required to determine what measure may be the most reflective of biological state in elephants, including analyzing concentrations around specific events, to determine if acute and/or chronic changes are related to physiological or mental status. Considering the complexity of both the hypothalamic–pituitary–adrenal (HPA) axis and immune response to stressors, it is feasible that single time-points may not be fully reflective of underlying physiology, and longitudinal analyses will provide useful insight into the relationships between these two physiological biomarkers. In a study by Tress and colleagues (2006), it was determined that to gain a reasonable representation of individual IgA concentrations, four fecal samples per individual were required, collected on 2 consecutive days, 28 days apart. This allowed for identification of dogs with consistently low fecal IgA concentrations, despite high intra-individual variability. Based on the observed variability in the current study, particularly for measures of secretory IgA, single samples likely will not be sufficient to use IgA concentrations as a measure of overall well-being in elephants.

Previous research in other species has suggested a negative correlation exists between IgA and GCs, including salivary measures in humans (Hucklebridge *et al.*, 1998) and dogs (Skandakumar *et al.*, 1995), and fecal measures in reindeer (Yin *et al.*, 2015). However, in many of these cases, data were obtained from single or duplicate samples per individual, as opposed to the longitudinal approach used here. Where repeated samples have been taken over a number of weeks in the past, for example during the acclimatization of mice to different cage types and social groupings, no correlation was apparent between fecal IgA and GC metabolites (Bundgaard *et al.*, 2012). This may be a reflection of the duration of the stressor; it has been suggested that IgA may be a useful biomarker of long-term stress (Valdimarsdottir and Stone, 1997), whereas HPA activity may be more appropriate for acute stressors (Royo *et al.*, 2004). Indeed, Tsujita and Morimoto (1999) suggested that salivary IgA can be a useful marker of welfare if the delayed effect of chronic stress is considered separately from the immediate effect of acute stress on this measure. However, with the exception of changes during cases of illness in two elephants, it should be noted that we did not assess the response to specific stressors in this study.

Primarily an immune protein, IgA can be highly responsive to health status, typically with decreased concentrations reflective of chronic pathology. Selective IgA deficiency is the most common form of primary immunodeficiency in humans (Cunningham-Rundles, 2001), and is associated with chronic gastrointestinal disease in both humans (Petty *et al.*, 1979) and dogs (Maeda *et al.*, 2013), where individuals with inflammatory bowel disease had significantly decreased concentrations of fecal IgA compared to healthy controls (Maeda *et al.*, 2013). The data from elephant E demonstrated short-term changes in excretion coincided with a severe systemic illness. In that case, both fecal IgA and GCs increased significantly, peaking at concentrations around 30- and 4-fold higher than baseline, respectively. Unfortunately, during the illness of elephant E, saliva, serum and urine were not collected, precluding us from determining whether this response would be evident in all sample types. This increase could indicate a physiological response to an acute stressor (Jarillo-Luna *et al.*, 2015) or an immune response to the pathology. Similarly, although from a shorter and less severe illness, data from female A further suggests that increases in IgA and GCs may be reflective of underlying health issues. Indeed the difference in magnitude of the responses observed in these two females could be reflective of the type or severity of their underlying condition. However, additional research is necessary to investigate this relationship further, determine if health issues that do not include gastrointestinal signs would invoke a similar response, and whether the inappetance that occurred in these two cases will have impacted gut-transit time or fecal composition, and the effect that may subsequently have on fecal IgA concentrations.

Results of this study highlight the importance of understanding differing response mechanisms when using IgA as a welfare indicator—chronic stressors may result in immune suppression and reductions in IgA, but acute illness also may be associated with increases in IgA concentration as part of an immune response to cope with underlying pathology. Thus, interpretation of IgA measures, like GCs, may not always be straightforward. Both IgA and GCs have been shown to increase in response to acute stressors of a non-immune nature (Tsujita and Morimoto, 1999; Jarillo-Luna *et al.*, 2015), and this certainly warrants further investigation before increased IgA concentrations can be considered a positive welfare indicator. As with other potential indicators of well-being, it is important to understand normal variation in physiological biomarkers both within and between individuals, as well as in response to specific events. Biomarkers must be put into context, incorporating longitudinal measurements of multiple indicators, such as IgA alongside GCs, to delineate concentrations indicative of an acute immune response or stressor, compared to those associated with longer-term positive or negative welfare states. The methodology described here provides a robust technique to investigate IgA in elephants, and these data provide a necessary baseline to interpret future data alongside other health and well-being measures, to determine whether incorporating IgA measurements will provide useful insight into elephant welfare.
